# Functional and structural analyses of *N*-acylsulfonamide-linked dinucleoside inhibitors of RNase A

**DOI:** 10.1111/j.1742-4658.2010.07976.x

**Published:** 2011-02

**Authors:** Nethaji Thiyagarajan, Bryan D Smith, Ronald T Raines, K Ravi Acharya

**Affiliations:** 1 Department of Biology and Biochemistry, University of BathUK; 2 Department of Biochemistry, University of Wisconsin–MadisonUSA; 3 Department of Chemistry, University of Wisconsin–MadisonUSA

**Keywords:** crystal structure, *N*-acylsulfonamide-linked dinucleoside inhibitors, RNase A

## Abstract

Molecular probes are useful for both studying and controlling the functions of enzymes and other proteins. The most useful probes have high affinity for their target, along with small size and resistance to degradation. Here, we report on new surrogates for nucleic acids that fulfill these criteria. Isosteres in which phosphoryl [R–O–P(O_2_^−^)–O–R′] groups are replaced with *N*-acylsulfonamidyl [R–C(O)–N^−^–S(O_2_)–R′] or sulfonimidyl [R–S(O_2_)–N^−^–S(O_2_)–R′] groups increase the number of nonbridging oxygens from two (phosphoryl) to three (*N*-acylsulfonamidyl) or four (sulfonimidyl). Six such isosteres were found to be more potent inhibitors of catalysis by bovine pancreatic RNase A than are parent compounds containing phosphoryl groups. The atomic structures of two RNase A·*N*-acylsulfonamide complexes were determined at high resolution by X-ray crystallography. The *N*-acylsulfonamidyl groups were observed to form more hydrogen bonds with active site residues than did the phosphoryl groups in analogous complexes. These data encourage the further development and use of *N*-acylsulfonamides and sulfonimides as antagonists of nucleic acid-binding proteins.

**Database:**

Structural data for the two RNase A complexes are available in the Protein Data Bank under accession numbers 2xog and 2xoi

## Introduction

Upon catalyzing the cleavage of RNA, RNases operate at the crossroads of transcription and translation. Bovine pancreatic RNase A (EC 3.1.27.5) is the best characterized RNase. A notoriously stable enzyme, RNase A retains its catalytic activity at temperatures near 100 °C or in otherwise denaturing conditions [1], and has numerous interesting homologs [2–4]. In humans, angiogenin (RNase 5) is an inducer of neovascularization, and plays an important role in tumor growth [5]. Eosinophil-derived neurotoxin (RNase 2) and eosinophil cationic protein (RNase 3) have antibacterial and antiviral activities. An amphibian homolog, onconase, has antitumor activity with clinical utility [6]. Even secretory RNases from the zebrafish share the RNase A scaffold [7]. Small-molecule inhibitors of these RNases could be used to investigate their broad biological functions.

The affinity of RNase A for RNA derives largely from hydrogen bonds [8], especially with the active site residues [9] and nucleobase [10]. The most potent small-molecule inhibitors of RNase A closely resemble RNA [11–17], and likewise form numerous hydrogen bonds with the enzyme. Pyrophosphoryl groups have four nonbridging oxygens, providing more opportunity for the formation of hydrogen bonds than is possible with a phosphoryl group. Accordingly, 5′-diphosphoadenosine 3′-phosphate and 5′-diphosphoadenosine 2′-phosphate exhibit strong affinity for RNase A [18], owing to extensive hydrogen-bonding interactions [19]. Pyrophosphoryl groups, however, have five rather than three backbone atoms. We reasoned that isosteres with additional nonbridging oxygen atoms but only three backbone atoms could be advantageous.

Much recent work has employed sulfur as the foundation for nucleoside linkers with multiple nonbridging oxygens. For example, achiral linkages have been made with a sulfone [R–S(O_2_)–R′] [20], sulfonate ester [R–S(O_2_)–O–R′] [21,22], sulfonamide [R–S(O_2_)–NH–R′] [23], sulfamate [R–O–S(O_2_)–NH–R′] [24], sulfamide [R–NH–S(O_2_)–NH–R′] [25,26], and *N*-acylsulfamate [R–O–S(O_2_)–NH–C(O)–R′] [27]. Of these functional groups, only the *N*-acylsulfamyl group has more nonbridging oxygens than does a phosphoryl group, but its length – four backbone atoms – compromises its utility as a surrogate.

We were intrigued by sulfonamides because of the relatively high anionicity of their nonbridging oxygens. Sulfonamide-linked nucleosides were employed first in antisense technology, where they were found to be highly soluble, and resistant to both enzyme-catalyzed and nonenzymatic hydrolysis [28,29]. Unlike this previous study, however, we chose to examine sulfonamides that were modified on nitrogen to install additional nonbridging oxygens.

We began our work by assessing the affinity of RNase A for two nucleic acid mimics that contain sulfonimide linkers [R–S(O_2_)–NH–S(O_2_)–R′], which have four nonbridging oxygens. We compared these mimics to a parent molecule that contains canonical phosphate linkers. Then, we assessed two mononucleosides and two dinucleosides containing an *N*-acylsulfonamide linker [R–S(O_2_)–NH–C(O)–R′], which has three nonbridging oxygens, in the place of a phosphoryl group. Finally, we determined the crystal structures of two *N*-acylsulfonamide-linked dinucleosides in complexes with RNase A. Together, our data lead to comprehensive conclusions regarding a new class of surrogates for the phosphoryl group.

## Results and Discussion

### Sulfonimides as inhibitors of RNase A

**Fig. 1:**
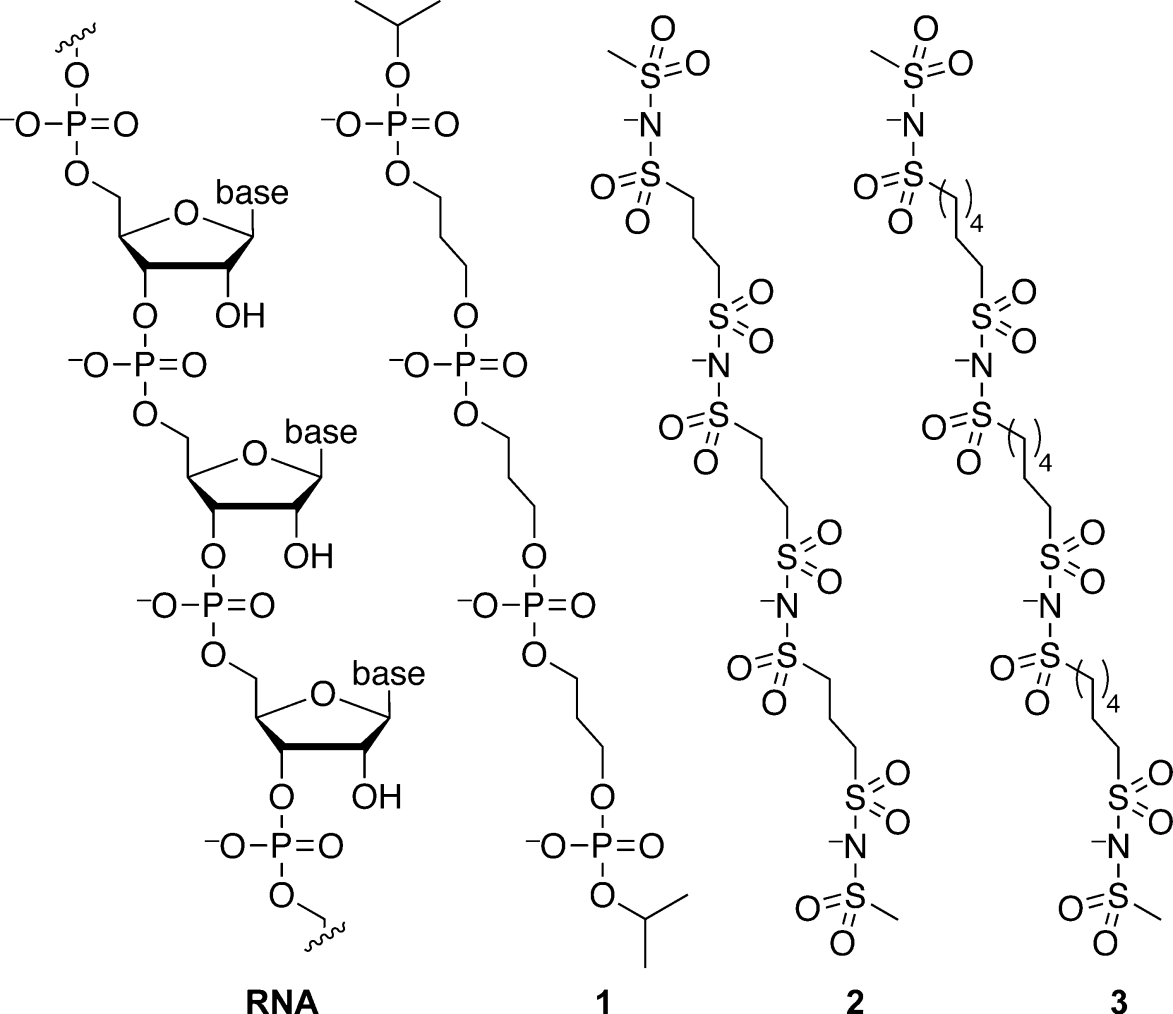
Chemical structures of RNA, tetraphosphodiester **1**, and tetrasulfonimides **2** and **3**.

We began by determining the ability of three backbone analogs of RNA to inhibit catalysis by RNase A. These analogs have a simple polyanionic backbone with neither a ribose moiety nor a nucleobase (Fig. 1). In tetraphosphodiester **1**, three carbon atoms separate the phosphoryl groups, mimicking the backbone of RNA but without the torsional constraint imposed by a ribose ring. To reveal a contribution from additional nonbridging oxygen atoms on enzyme inhibition, we used tetrasulfonimide **2**, which has three carbon atoms between its sulfonimidyl groups, and tetrasulfonimide **3**, which has six.

Under no-salt conditions, which encourage Coulombic interactions, we could only set a lower limit of *K*_i_ > 10 mm for tetraphosphodiester **1** (Table 1). Previously, we reported that RNase A binds to a tetranucleotide containing four phosphoryl groups with *K*_d_ = 0.82 μm under low-salt conditions [30]. Thus, we conclude that the ribose moiety and nucleobase of a nucleic acid increase its affinity for RNase A by > 10^4^-fold.

**Table 1:** Constants for inhibition of RNase A catalysis by compounds **1**–**7**

Compound	*K*_i_ (mm), no salt^a^	*K*_i_ (mm), 0.10 m salt^b^
Tetraphosphodiester **1**	> 10	ND
Tetrasulfonimide **2**	0.11 ± 0.02	8.3 ± 1.7
Tetrasulfonimide **3**	0.33 ± 0.07	∼ 10
*N*-acylsulfonamide **4**	ND	5.3 ± 0.5
*N*-acylsulfonamide **5**	ND	4.8 ± 0.3
*N*-acylsulfonamide **6**	ND	0.46 ± 0.03
*N*-acylsulfonamide **7**	ND	0.37 ± 0.01

a Values (±standard error) in 0.05 m Bistris/HCl buffer at pH 6.0.

b Values (±standard error) in 0.05 m Mes/NaOH buffer at pH 6.0, containing NaCl (0.10 m).

Then, we found that tetrasulfonimide **2** inhibits catalysis by RNase A with *K*_i_ = 0.11 mm under no-salt conditions (Table 1). Apparently, the additional nonbridging oxygens of tetrasulfonimide **2** provide > 10^2^-fold greater affinity for RNase A. In the presence of 0.10 m NaCl, the *K*_i_ value of tetrasulfonimide **2** increased by 80-fold, indicating that binding had a Coulombic component [31,32]. This finding is consistent with RNase A (pI 9.3) [33] being cationic and each sulfonimidyl group (N–H p*K*_a_ = −1.7) [34] being anionic in aqueous solution.

Finally, we found that tetrasulfonimide **3** inhibits catalysis with *K*_i_ = 0.33 ± 0.07 mm under no-salt conditions (Table 1). The slightly weaker affinity of tetrasulfonimide **3** than of tetrasulfonimide **2** is consistent with the spacing of their sulfonimidyl groups. RNase A has four well-defined phosphoryl group-binding subsites [35,36]. The spacing of the sulfonimidyl groups in tetrasulfonimide **2** is analogous to that of the phosphoryl groups in a nucleic acid (Fig. 1), and these sulfonimidyl groups are poised to occupy the enzymatic subsites for phosphoryl groups. In comparison, the separation between the sulfonimidyl groups in tetrasulfonimide **3** is too large.

### *N*-Acylsulfonamide-linked dinucleosides as inhibitors of RNase A

**Fig. 2:**
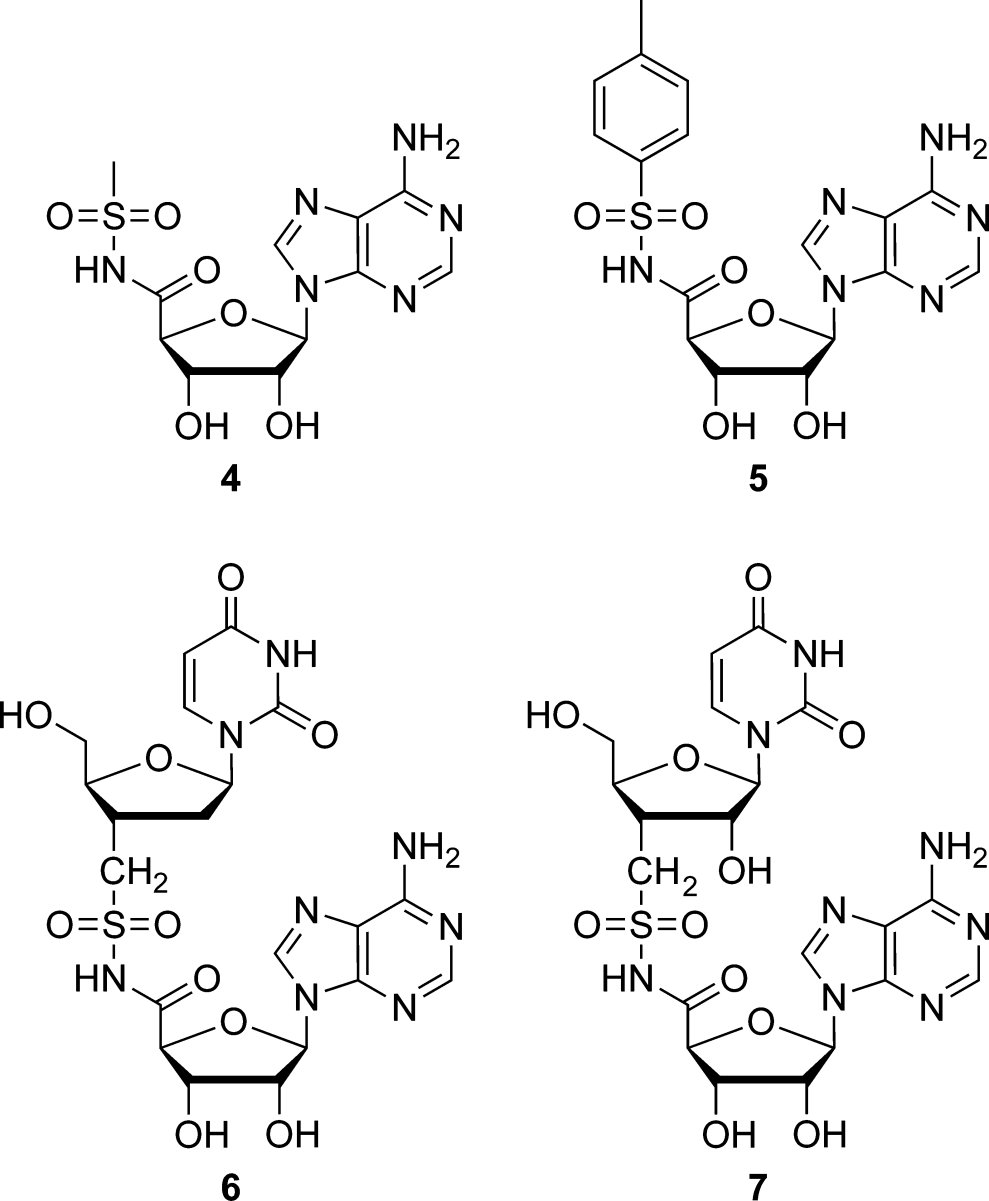
Chemical structures of *N*-acylsulfonamide-linked nucleosides **4**–**7**.

**Fig. 3:**
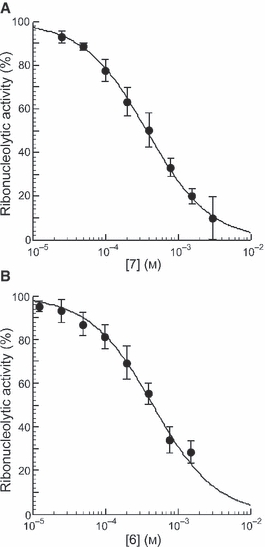
Isotherms for the binding of *N*-acylsulfonamide-linked dinucleosides to RNase A. Data were fitted to Eqn (1). (A) *N*-acylsulfonamide **7**, *K*_i_ = (3.7 ± 0.1) × 10^−4^ m. (B) *N*-acylsulfonamide **6**, *K*_i_ = (4.6 ± 0.3) × 10^−4^ m.

Given the efficacy of the sulfonimidyl group as a phosphoryl group surrogate, we sought to determine the advantage of adding nonbridging oxygens to a nucleic acid. To do this, we employed an *N*-acylsulfonamidyl group, which has three nonbridging oxygen atoms and is anionic (N–H p*K*_a_ = 4–5) [34]. In compounds **4**–**7** (Fig. 2; Fig. S1), an *N*-acylsulfonamidyl group replaces the phosphoryl group in AMP or uridylyl(3′→5′)adenosine (UpA). We found that each of these compounds inhibited catalysis by RNase A more than did tetrasulfonimide **2** or tetrasulfonimide **3**, which are not nucleosides (Fig. 3; Table 1). The two AMP analogs inhibited RNase A with *K*_i_ values of ∼ 5 mm. In contrast, AMP itself has a *K*_i_ of 33 mm [37]. The two UpA analogs inhibited RNase A with *K*_i_ values of ∼ 0.4 mm (Table 1). In contrast, thymidylyl(3′→5′)2′-deoxyadenosine inhibits RNase A with *K*_i_ = 1.2 mm [9]. We conclude that replacing a single phosphoryl group with an *N*-acylsulfonamidyl group confers an approximately five-fold increase in affinity for RNase A.

Of compounds **1**–**7**, RNase A binds most tightly with *N*-acylsulfonamides **6** and **7**. These inhibitors closely mimic a natural substrate for RNase A, UpA [38,39], which is cleaved by the enzyme with a rate enhancement of nearly a trillion-fold [40]. Accordingly, we decided to investigate their interactions with RNase A in detail by using X-ray crystallography.

### Three-dimensional structures of RNase A·*N*-acylsulfonamide-linked nucleoside complexes

**Fig. 4:**
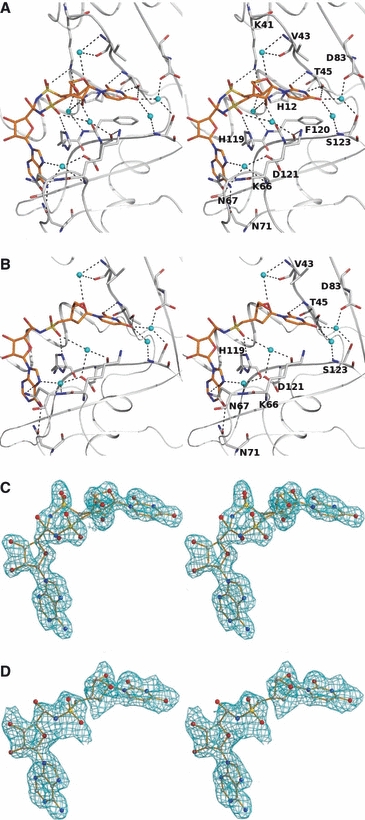
(A, B) Schematic and stereo representation of hydrogen bonds in the RNase A complex with *N*-acylsulfonamide **7** and *N*-acylsulfonamide **6**, respectively. *N*-Acylsulfonamide **7** and *N*-acylsulfonamide **6**, gold; active site residues, pea-green; RNase A, gray. Hydrogen bonds are represented as dashed lines, and water molecules are in cyan. (C, D) Stereo pictures of 2*F*_o_ − *F*_c_ contoured at 1.0*σ* for *N*-acylsulfonamide **7** and *N*-acylsulfonamide **6**, respectively.

The three-dimensional structures of *N*-acylsulfonamides **6** and **7** in complex with RNase A were determined by X-ray crystallography (Table 2). The structures were solved to a resolution of 1.72 Å by molecular replacement in a centered monoclinic (*C*2) space group with two molecules per asymmetric unit. *N*-Acylsulfonamides **6** and **7** (Fig. 2) bound at the active site of RNase A are more fully observed in molecule A (Fig. 4). In molecule B, only adenine nucleosides are apparent (an observation similar to those made with RNase A–inhibitor complexes reported previously by us in this space group). Alternative conformations for some parts of *N*-acylsulfonamide **7**, highlighting the flexibility around the ribose moieties, are observed and are built into the structure. A similar alternative conformation was not observed for *N*-acylsulfonamide **6**.

**Table 2:** X-ray data collection and refinement statistics. *R*_symm_ = Σ_*h*_ Σ_*i*_ |*I*(*h*) − *I*_*i*_(*h*)|/Σ_*h*_ Σ_*i*_*I*_*i*_(*h*), where *I*_*i*_(*h*) and *I*(*h*) are the *i*th and the mean measurements of the intensity of reflection *h*, respectively. *R*_cryst_ = Σ_*h*_ |*F*_o_ − *F*_c_|/Σ_*h*_*F*_o_, where *F*_o_ and *F*_c_ are the observed and calculated structure factor amplitudes of reflection *h*, respectively. *R*_free_ is equal to *R*_cryst_ for a randomly selected 5.0% subset of reflections not used in the refinement

	RNase A·*N*-acylsulfonamide **7**	RNase A·*N*-acylsulfonamide **6**
Space group	*C2*	*C2*
Cell dimensions	*a* = 101.0 Å	*a* = 101.0 Å
	*b* = 33.1 Å	*b* = 33.2 Å
	*c* = 72.6 Å	*c* = 72.8 Å
	*α* = *γ* = 90°	*α* = *γ* = 90°
	*β* = 90.4°	*β* = 90.9°
Resolution range (Å)	50–1.72	50–1.72
*R*_symm_ (outer shell)	0.060 (0.171)	0.062 (0.192)
*I*/*σI* (outer shell)	17.5 (6.0)	17.2 (5.7)
Completeness (outer shell) (%)	98.5 (94.5)	98.0 (92.7)
Total no. of reflections	174 818	186 775
Unique no. of reflections	26 158	26 200
Redundancy (outer shell)	3.0 (2.8)	3.1 (2.9)
Wilson *B*-factor (Å^2^)	17.8	18.1
*R*_cryst_/*R*_free_	0.212/0.246	0.214/0.244
Average *B*-factor (Å^2^)		
Overall	18.1	18.3
Protein (chain A, B)	16.2, 16.5	16.4, 16.3
Ligand	21.8, 56.0	34.2, 43.2
Solvent	26.5	25.6
rmsd		
Bond length (Å)	0.007	0.007
Bond angle (°)	1.439	1.113
PDB codes	2xog	2xoi

*N*-Acylsulfonamide **6** (2′-deoxy) and *N*-acylsulfonamide **7** (2′-oxy) differ by only one atom. These two dinucleotide isosteres adopt a similar conformation upon binding to RNase A, and occupy the same enzymic subsites as do the dinucleotides cytidylyl(3′→5′)adenosine [Protein Data Bank (PDB) code 1r5c] [41] and UpA (PDB code 11ba) [42]. The structure of *N*-acylsulfonamide **7** was refined with full occupancy, except for the alternative conformations observed for the *N*-acylsulfonamidyl group and the addition of O_2_′. The value of the nucleoside torsion angle *χ* (Table S1) indicates that the compounds are bound in an *anti* conformation, which is the preferred orientation for bound adenine and pyrimidines [43]. The two ribose moieties exhibit a high degree of flexibility, as expected. The backbone torsion angle *δ* for the bound ribose units is in an unfavorable conformation, representing neither a bound nor an unbound state, although the *γ* torsion angle represents the bound state for ribose units with *±sc*. In *N*-acylsulfonamide **7**, the *γ* torsion angle for the ribose of adenine exhibits an unfavorable *+ac* puckering in one of its alternative conformations.

**Fig. 5:**
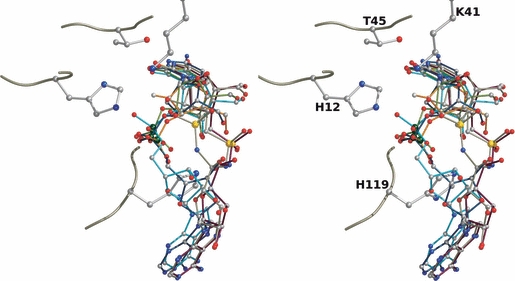
Superposition (stereo representation) of *N*-acylsulfonamide **6** (gray) and *N*-acylsulfonamide **7** (maroon) (this work) on uridylyl(2′→5′)adenosine (cyan), cytidine 2′-phosphate (green), 2′-deoxycytidylyl(3′→5′)2′-deoxyadenosine (blue), and 2′-fluoro-2′-deoxyuridine 3′-phosphate (gold) (PDB codes: 11ba, 1jvu, 1r5c, and 1w4q, respectively). Sulfur atoms are in yellow; phosphorus atoms are in forest green.

The pseudorotation angles for the uridine of *N*-acylsulfonamide **7** were found in both the C_3_′-*endo* (*N*) conformation and the O_4_′-*endo* conformation, whereas the C_3_′-*endo* conformation was preferred for *N*-acylsulfonamide **6**. C_3_′-*endo* puckering had been observed previously for bound uridylyl(2′→5′)adenosine [42], 2′-CMP [44], and diadenosine 5′,5′′,5′′′-*P*′,*P*′′,*P*′′′ triphosphate (Ap_3_A) [17]. Solution NMR studies have shown that the C_3_′-*endo* puckering is a predominant state for unbound furanose rings [44,45]. O_4_′-*endo* puckering is an unusual conformation, and was observed in the complexes of RNase A with 2′-fluoro-2′-deoxyuridine 3′-phosphate [11] and Ap_3_A [17] (Fig. 5).

### Hydrogen bonding in RNase A·*N*-acylsulfonamide-linked nucleoside complexes

The hydrogen-bonding pattern exhibited by the nucleobases is conserved in both the 2′-oxy (**7**) and 2′-deoxy (**6**) *N*-acylsulfonamides (Table S2). In both structures, the bound inhibitors span the nucleobase-binding subsites. Surprisingly, however, the *N*-acylsulfonamidyl groups point away from the active site (Figs 4 and 5). In *N*-acylsulfonamide **7**, O_2*S*_ of the *N*-acylsulfonamidyl group forms hydrogen bonds with active site residues His119 and Asp121 (mediated by a water molecule). In one of its alternative states, O_1*S*_ of the *N*-acylsulfonamidyl group forms a hydrogen bond with Lys41. In *N*-acylsulfonamide **6**, where only a single conformation was observed for the bound *N*-acylsulfonamidyl group, O_2*S*_ forms two hydrogen bonds with His119 and Asp121 (mediated by a water molecule). Thus, replacing a phosphoryl group with an *N*-acylsulfonamidyl group leads to new hydrogen-bonding interactions.

RNase A cleaves UpA and UpG uridylyl(3′→5′)guanosine (UpG) with similar *K*_m_ values but significantly different *k*_cat_ values [46]. The similarity in the *K*_m_ values is attributable to the uracil moiety binding in the same fashion [38], which could trigger the initial binding of both substrates. In UpG, the binding of the guanine moiety is deterred by exocyclic O_6_. Close inspection shows that the relevant subsite of RNase A has a negative potential and hence cannot accommodate an electronegative atom. In contrast, the exocyclic N_6_-amino group of adenine forms a hydrogen bond with the side chain of Asn71, increasing the affinity of RNase A for UpA. This hydrogen bond is apparent in the complexes with *N*-acylsulfonamides **6** and **7** (Table S2; Fig. 4).

In all reported RNase A·nucleotide complexes, at least one atom of ribose (either O_2_′ or O_3_′) appears to interact intimately with the enzyme. The ribose unit of uridine in *N*-acylsulfonamide **7** forms four hydrogen bonds. O_4_′ shares two hydrogen bonds with the enzyme, and O_2_′ forms two additional hydrogen bonds in each of its conformations. Thus, in either observed conformation of *N*-acylsulfonamide **7**, there are a total of four hydrogen bonds formed by the uridine ribose. Of the two hydrogen bonds exhibited by these two atoms, one is a direct interaction with the enzyme and the other is mediated by a water molecule. In the complex with *N*-acylsulfonamide **6**, which lacks an O_2_′, only O_4_′ of the uridine ribose forms hydrogen bonds with the enzyme. O_5_′ of the adenosine ribose forms a hydrogen bond with active site residue His119 in its alternative form in *N*-acylsulfonamide **7**.

Overall, *N*-acylsulfonamide **7** and *N*-acylsulfonamide **6** exhibit 12(12) and 8(11) hydrogen bonds with RNase A (including solvent-mediated interactions in parentheses), respectively (Table S2). These numbers are comparable to those in the complexes with uridylyl(2′→5′)adenosine [10(5)] [42], 3′-CMP [11(2)] [46], and 2′-deoxycytidylyl(3′→5′)2′-deoxyadenosine [10(5)] [47]. Thus, replacing a phosphoryl group with an *N*-acylsulfonamidyl group can recapitulate, or even enhance, the characteristic structural interactions of a nucleic acid with a protein.

## Conclusions

The functional and structural studies presented herein demonstrate the attributes of *N*-acylsulfonamidyl and sulfonimidyl groups as surrogates for the phosphoryl groups of nucleic acids. The structural complexes of two *N*-acylsulfonamide-linked nucleosides with RNase A closely mimic the binding by nucleic acids. The attributes and versatility of *N*-acylsulfonamidyl and sulfonimidyl groups are ripe for exploitation in the creation of nucleic acid surrogates.

## Experimental procedures

A fluorogenic RNase substrate, 6-FAM–dArUdAdA–6-TAMRA (where 6-FAM is a 6-carboxyfluorescein group at the 5′-end and 6-TAMRA is a 6-carboxytetramethylrhodamine group at the 3′-end), was from Integrated DNA Technologies (Coralville, IA, USA). RNase A from Sigma Chemical (St. Louis, MO, USA) was used for crystallization and structure determination of RNase A·sulfonamide complexes. RNase A produced by heterologous expression [48] was used in assays to determine *K*_i_ values. All other chemicals and biochemicals were of reagent grade or better, and were used without further purification.

Compounds **1**–**3** [49,50] and **4**–**7** [51] were synthesized as described previously, and were generous gifts from T. S. Widlanski, B. T. Burlingham, and D. C. Johnson, II (Indiana University, USA).

### Determination of *K*_i_ values

Compounds **1**–**7** were assessed as inhibitors of catalysis of 6-FAM–dArUdAdA–6-TAMRA cleavage by RNase A [52,53]. Briefly, assays were performed in 2.00 mL of either 0.05 m Bistris/HCl buffer at pH 6.0 or 0.05 m Mes/NaOH buffer at pH 6.0, containing NaCl (0.10 m) that also contained 6-FAM–dArUdAdA–6-TAMRA (0.06 μm) and RNase A (1–5 pm). Mes was purified prior to use to remove inhibitory contaminants, as described previously [54]. Fluorescence (*F*) was measured with 493 and 515 nm as the excitation and emission wavelengths, respectively, using a QuantaMaster 1 Photon Counting Fluorometer equipped with sample stirring (Photon Technology International, South Brunswick, NJ, USA). The Δ*F*/Δ*t* value was measured for 3 min after the addition of RNase A. An aliquot of the putative competitive inhibitor (I) dissolved in the assay buffer was added, and Δ*F*/Δ*t* was recorded for 3 min. The concentration of I was doubled repeatedly at 3-min intervals. Excess RNase A was then added to the mixture to ensure that < 10% of the substrate had been cleaved prior to completion of the inhibition assay. Apparent changes in ribonucleolytic activity caused by dilution were corrected by comparing values with those from an assay in which aliquots of buffer were added. Values of *K*_i_ for competitive inhibition were determined by nonlinear least squares regression analysis of data fitted to Eqn (1), where (Δ*F*/Δ*t*)_0_ was the activity prior to the addition of inhibitor.
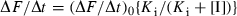
(1)

### X-ray crystallography

Crystals of RNase A were grown by using the hanging drop vapor diffusion method [19]. Crystals of RNase A·*N*-acylsulfonamide complexes were obtained by soaking crystals in the inhibitor solution containing mother liquor [0.02 m sodium citrate buffer at pH 5.5, containing 25% (w/v) poly(ethylene glycol) 4000]. Diffraction data for the two complexes were collected at 100 K, with poly(ethylene glycol) 4000 (30% w/v) as a cryoprotectant, on station PX 9.6 at the Synchrotron Radiation Source (Daresbury, UK), using a Quantum-4 CCD detector (ADSC Systems, Poway, CA, USA). Data were processed and scaled in space group *C*2 with the hkl2000 software suite [55]. Initial phases were obtained by molecular replacement, with an unliganded RNase A structure (PDB code 1afu) as a starting model. Further refinement and model building were carried out with refmac [56] and coot [57], respectively (Table 2). With each data set, a set of reflections (5%) was kept aside for the calculation of *R*_free_ [58]. The *N*-acylsulfonamide inhibitors were modeled with 2*F*_o_ − *F*_C_ and *F*_o_ − *F*_C_sigmaa-weighted maps. The ligand dictionary files were created with the sketcher tool in the ccp4i interface [59]. All structural diagrams were prepared with bobscript [60].

## Abbreviations

PDB: Protein Data Bank
UpA: uridylyl(3′→5′)adenosine

## Supporting information

The following supplementary material is available:

**Fig. S1.** Atom numbering for compounds **6** and **7**.

**Table S1.** Torsion angles of nucleosides in RNase A·*N*-acylsulfonamidelinked nucleoside complexes.

**Table S2.** Putative hydrogen bonds in RNase A·*N*-acylsulfonamide-linked nucleoside complexes.

This supplementary material can be found in the online version of this article.

Please note: As a service to our authors and readers, this journal provides supporting information supplied by the authors. Such materials are peer-reviewed and may be re-organized for online delivery, but are not copy-edited or typeset. Technical support issues arising from supporting information (other than missing files) should be addressed to the authors.
