# Supplementary material for: Functional and structural analyses of N-acylsulfonamide-linked dinucleoside inhibitors of RNase A
Source: FEBS J. 2011 Feb;278(3):541–9. doi: 10.1111/j.1742-4658.2010.07976.x (PMC3039443; doi:10.1111/j.1742-4658.2010.07976.x)
Supplement: Supplementary file 1 [file febs0278-0541-SD1.pdf]

the FEBS Journal

www.febsjournal.org

Functional and structural analyses of *N*-acylsulfonamide-linked dinucleoside inhibitors of RNase A

Nethaji Thiyagarajan, Bryan D. Smith, Ronald T. Raines and K. Ravi Acharya

DOI: 10.1111/j.1742-4658.2010.07976.x

Supplementary Material (Thiyagarajan *et al*)

Figure SF1: Atom numbering for compounds 6 and 7

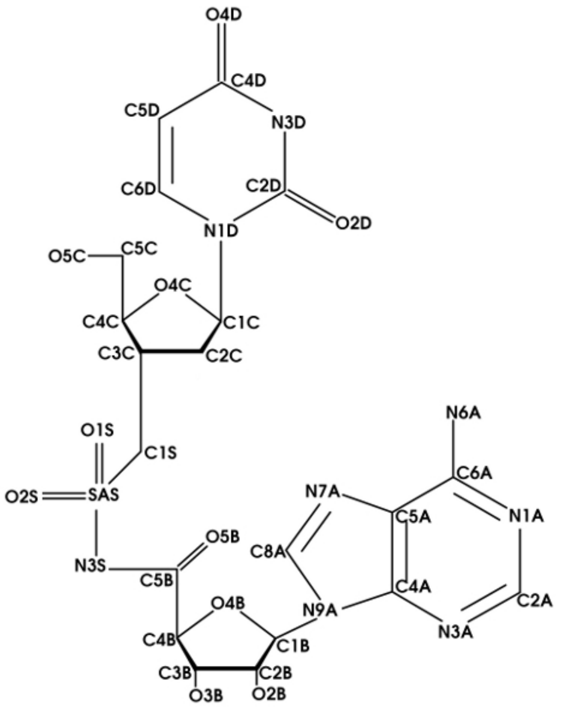

6

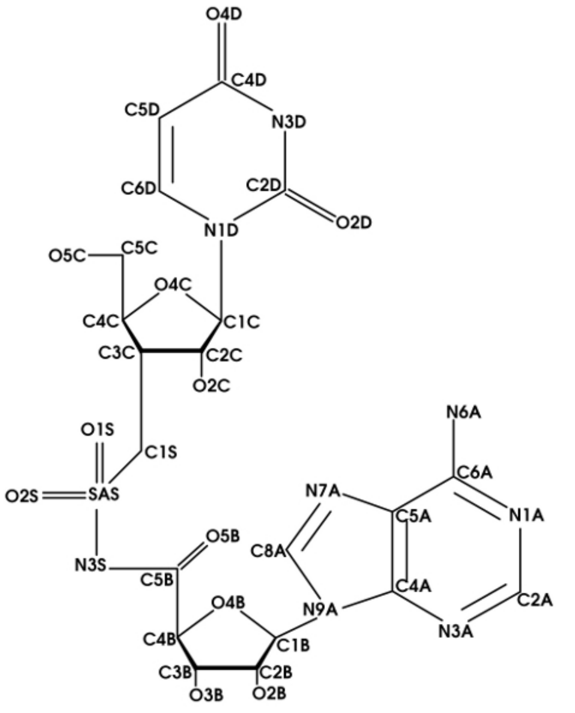

7

Table ST1: Torsion angles of nucleosides in RNase A·*N*-acylsulfonamide-linked nucleoside complexes. The atom numbering listed are as shown in the Figure SF1 above.

	N-acylsulfonamide **7**	*N*-acylsulfonamide **6**
Backbone torsion angles		
C1B-C2B-C3B-O3B	118.5	121
C2B-C3B-C4B-C5B	-133.3	-141.6
O3B-C3B-C4B-C5B (δ)	107.2 (+*ac*)	98.4 (+*ac*)
O4B-C4B-C5B-O5BA*	-174.3	-157
O4B-C4B-C5B-O5BB*	-18.7	—
O2B-C2B-C3B-O3B	0.0	2.5
C1B-O4B-C4B-C5B	146.9	154.7
C3B-C4B-C5B-O5BA* (γ)	-55.7 (-*sc*)	-37.1 (-*sc*)
C3B-C4B-C5B-O5BB* (γ)	99.8 (+*ac*)	—
O3B-C3B-C4B-O4B	-132.7	-139
O4B-C1B-C2B-O2B	135.0	136.4
C3C-C4C-C5C-O5C (γ)	50.6 (+*sc*)	59.1 (+*sc*)
O5C-C5C-C4C-O4C	-65.0	-60
C5C-C4C-O4C-C1C	136.7	134.8
O4C-C1C-C2C-O2CA*	-114.5	—
O4C-C1C-C2C-O2CB*	-165.6	—
C2C-C3C-C4C-C5C	-140.1	-137.9
Glycosyl torsion angles		
C4A-N9A-C1B-O4B (χ')	-125.5 (-*ac*)	-132.8 (-*ac*)
C2D-N1D-C1C-O4C (χ')	-131.3 (-*ac*)	-121.4 (-*ac*)
Pseudorotation angles		
C4B-O4B-C1B-C2B (υ_0)	-24.8	-29.4
C4C-O4C-C1C-C2CA* (υ_0)	6.1	-2.3
C4C-O4C-C1C-C2CB* (υ_0)	-26.3	-
O4B-C1B-C2B-C3B (υ_1)	15.4	16.4
C3C-C2CA*-C1C-O4C (υ_1)	-24.3	-8.5
C3C-C2CB*-C1C-O4C (υ_1)	26.3	-
C1B-C2B-C3B-C4B (υ_2)	-1.2	1.6
C1C-C2CA*-C3C-C4C (υ_2)	32.5	15.3
C1C-C2CB*-C3C-C4C (υ_2)	-17.9	-
C2B-C3B-C4B-O4B (υ_3)	-13.3	-19
C2CA*-C3C-C4C-O4C (υ_3)	-29.3	-17
C2CB*-C3C-C4C-O4C (υ_3)	3.0	—

C3B-C4B-O4B-C1B (υ_4)	23.9	30.5
C3C-C4C-O4C-C1C (υ_4)	14.8	12.1
Phase		
XXA[a]	89.6<br>$O_{4'}$-*endo*	87.0<br>$O_{4'}$-*endo*
XXU[a]	7.8, 89.7<br>$C_{3'}$-*endo*, $O_{4'}$-*endo*	25.9<br>$C_{3'}$-*endo*
N-Acylsulfonamide-linked nucleoside torsion angles		
O4B-C4B-C5B-N3SA*	-51.7	-37.8
O4B-C4B-C5B-N3SB*	103.0	—
C4B-C5B-N3SA*-SASA*	160.8	155
C4B-C5B-N3SB*-SASB*	-159.1	—
O5BA*-C5B-N3SA*-SASA*	-77.0	-84.9
O5BB*-C5B-N3SB*-SASB*	-44.8	—
C5B-N3SA*-SASA*-O1S	-68.8	76.9
C5B-N3SB*-SASB*-O1S	109.2	—
C5B-N3SA*-SASA*-O2SA*	-161.7	-164.8
C5B-N3SB*-SASB*-O2SB*	-75.8	—
N3SA*-SASA*-C1SA*-C3C	-175.2	-166
N3SB*-SASB*-C1SB*-C3C	-120.8	—
O1SA*-SASA*-C1SA*-C3C	68.3	73.8
O1SB*-SASB*-C1SB*-C3C	122.4	—
O2SA*-SASA*-C1SA*-C3C	-55.4	-46.6
O2SB*-SASB*-C1SB*-C3C	6.3	—
SASA*-C1SA*-C3C-C2CA*	159.9	142.3
SASB*-C1SB*-C3C-C2CB*	-153.5	—
SASA*-C1SA*-C3C-C4C	-67.8	-98.4
SASB*-C1SB*-C3C-C4C	-31.5	—
C1SA*-C3C-C2CA*-O2CA*	57.7	—
C1SB*-C3C-C2CB*-O2CB*	-18.5	—
C1SA*-C3C-C4C-C5C	75.2	99.9
C1SB*-C3C-C4C-C5C	122.6	—
C1SA*-C3C-C2CA-C1C	172.7	138.7
C1SB*-C3C-C2CB-C1C	109.4	—
C1SA*-C3C-C4C-O4C	-167.9	-139.1
C1SB*-C3C-C4C-O4C	-120.4	—

*Atom in alternative conformation with respect to *N*-acylsulfonamide **7**.

[a]XXA, ribose attached to adenine; XXU, ribose/deoxyribose attached to uridine.

<u>Table ST2:</u> Putative hydrogen bonds in RNase A·*N*-acylsulfonamide-linked nucleoside complexes. The ligand atom numbering listed are as shown in the Figure SF1 above.

Compound	Ligand atom	RNase A residue	Distance (Å)
N-acylsulfonamide **7**	O2CA*	Lys41-NZ<br>Water–Phe120-N, His12-NE2	2.78<br>3.29–2.91, 2.71
	O2SA*	His119-NE2<br>Water–Asp121-O	3.47<br>3.48–2.80
	N6A	Asn71-OD1	3.01
	N1A	Asn71-ND2	3.12
	N7A	Asn67-ND2<br>Water–Lys66-N, Asn67-N, Asp121-OD1	3.24<br>3.00–2.97, 2.80
	O5BB*	His119-NE2	3.33
	O1SB*	Lys41-NZ	3.37
	O2CB*	Phe120-O<br>Water–Phe120-N, His12-NE2	2.52<br>3.04–2.91, 2.71
	O4C	Val43-O<br>Water–Val43-N, Val43-O	3.17<br>3.05–3.05, 2.81
	O2D	Thr45-N	2.95
	N3D	Thr45-OG1	2.72
	O4D	Thr45-OG1<br>Water–Ser123-N<br>Water–Ser123-OG, Asp83-OD1	3.30<br>3.04–3.02<br>2.50–2.62, 2.71
N-acylsulfonamide **6**	N6A	Asn71-OD1	2.97
	N1A	Asn71-ND2	3.22
	N7A	Asn67-ND2<br>Water–Asn67-N, Lys66-N, Asp121-OD1	3.25<br>2.93–3.06, 3.49, 2.79
	O4C	Val43-O<br>Water–Val43-N, Val43-O	3.51<br>3.36–3.05, 2.94
	O2S	His119-NE2<br>Water–Asp121-OD1, Asp121-O	3.27<br>3.43–3.14, 2.72
	N3D	Thr45-OG1	2.75
	O2D	Thr45-N	2.96
	O4D	Thr45-OG1<br>Water–Asp83-OD1, Ser123-OG<br>Water–Ser123-N, Ser123-O	3.41<br>2.59–2.75, 2.63<br>3.06–2.97, 3.47

*Atom in alternative conformation.
